# Molecular Landscape and Diagnostic Model of MASH: Transcriptomic, Proteomic, Metabolomic, and Lipidomic Perspectives

**DOI:** 10.3390/genes16040399

**Published:** 2025-03-29

**Authors:** Yilong Chen, Shuixiu Bian, Jiamei Le

**Affiliations:** 1 School of Health Science and Engineering, University of Shanghai for Science and Technology, Shanghai 200093, China; diomaker@163.com (Y.C.); bianshuix@163.com (S.B.); 2 Shanghai Key Laboratory of Molecular Imaging, Zhoupu Hospital, Shanghai University of Medicine and Health Sciences, Shanghai 201318, China

**Keywords:** MASH, transcriptomics, proteomics, metabolomics, lipidomics, diagnostic model

## Abstract

Metabolic dysfunction-associated steatohepatitis (MASH), a progressive form of fatty liver disease, presents a significant global health challenge. Despite extensive research, fully elucidating its complex pathogenesis and developing accurate non-invasive diagnostic tools remain key goals. Multi-omics approaches, integrating data from transcriptomics, proteomics, metabolomics, and lipidomics, offer a powerful strategy to achieve these aims. This review summarizes key findings from multi-omics studies in MASH, highlighting their contributions to our understanding of disease mechanisms and the development of improved diagnostic models. Transcriptomic studies have revealed widespread gene dysregulation affecting lipid metabolism, inflammation, and fibrosis, while proteomics has identified altered protein expression patterns and potential biomarkers. Metabolomic and lipidomic analyses have further uncovered significant changes in various metabolites and lipid species, including ceramides, sphingomyelins, phospholipids, and bile acids, underscoring the central role of lipid dysregulation in MASH. These multi-omics findings have been leveraged to develop novel diagnostic models, some incorporating machine learning algorithms, with improved accuracy compared to traditional methods. Further research is needed to validate these findings, explore the complex interplay between different omics layers, and translate these discoveries into clinically useful tools for improved MASH diagnosis and prognosis.

## 1. Introduction

Non-alcoholic fatty liver disease (NAFLD) was recently renamed metabolic dysfunction-associated fatty liver disease (MASLD) [1]. Metabolic dysfunction-associated steatohepatitis (MASH) is a progressive form of MASLD and poses a major threat to global health. A recent meta-analysis showed that the global prevalence of NAFLD/MASLD is approximately 30%, the global prevalence of non-alcoholic steatohepatitis (NASH)/MASH is approximately 5.27%, and the prevalence in patients with type 2 diabetes (T2D) is even higher, reaching 34% [2,3]. Among them, the Middle East and North Africa are the regions with the highest prevalence of NAFLD/MASLD [4]. The complex pathogenesis of MASH forms a vicious cycle of liver damage, promotes disease progression, and aggravates the global health burden of MASH.

Clinically, diagnosing MASH and accurately staging fibrosis remain challenging [5]. Liver biopsy, while providing a definitive diagnosis, is invasive, while noninvasive methods such as transient elastography and serum biomarkers lack sufficient specificity and accuracy [6]. Critically, these conventional approaches fail to capture the multidimensional molecular perturbations underlying MASH pathogenesis, which involve dynamic interactions between genetic predisposition, metabolic dysregulation, and immune-inflammatory responses [7,8]. This limitation has driven the emergence of omics technologies as indispensable tools for MASH research [9]. Their value in MASH research lies in three core capabilities: parallel analysis of thousands of biomolecules, linkage of molecular signatures to pathophysiological processes, and derivation of clinically actionable biomarkers through advanced computational models [10].

Current reviews of multi-omics in MASLD/MASH have mainly focused on biomarker discovery, neglecting comprehensive investigations on how multi-omics technologies can be applied to unravel the complex pathophysiology and develop diagnostic models specifically for MASLD/MASH [11,12]. This review addresses this gap by deeply exploring the research of transcriptomics, proteomics, metabolomics, and lipidomics in MASH, highlighting key discoveries and advances in diagnosis, and outlining future research directions. This comprehensive perspective provides an overall understanding of the pathogenesis of MASH. By summarizing and analyzing key genes, targets, pathways, and diagnostic models identified through multi-omics research in the pathogenesis and progression of MASH, it offers important insights for clinical diagnosis, targeted drug development, and prognosis of MASH.

## 2. Pathophysiology of MASH

Disordered lipid metabolism is the initiating event in MASH pathogenesis [13]. The core defect is a disruption of hepatic lipid homeostasis, culminating in hepatic steatosis, the accumulation of triglycerides (TGs) within hepatocytes [14]. This lipid imbalance originates from increased dietary fat intake, heightened adipose tissue lipolysis due to insulin resistance (IR), and elevated hepatic de novo lipogenesis (DNL) [14]. IR, a central metabolic abnormality in MASH, simultaneously increases peripheral free fatty acid (FFA) supply to the liver and stimulates DNL within the liver [15]. As the liver’s capacity for FFA oxidation becomes overwhelmed, excess FFAs are shunted toward TG synthesis and storage [16]. The lipid accumulation in hepatocytes induces endoplasmic reticulum (ER) stress and oxidative stress, thereby triggering lipotoxicity [17,18]. This lipotoxic environment directly damages hepatocytes and activates inflammatory signaling pathways [18].

Inflammation in MASH is critically mediated by Kupffer cells (KCs), the liver-resident macrophages [19]. Hepatocyte injury and cellular stress, resulting from lipotoxicity, trigger KC activation [19]. Activated KCs polarize towards a pro-inflammatory M1 phenotype, becoming a major source of inflammatory mediators [20]. They release pro-inflammatory cytokines such as TNF-α, IL-1β, and IL-6, and chemokines like CCL2 [19]. These cytokines amplify local inflammation and, in conjunction with reactive oxygen species (ROS) produced by activated KCs, further exacerbate hepatocyte injury and oxidative stress [21]. This circulatory interaction results in persistent chronic inflammation and oxidative stress in the liver.

Hepatic fibrosis, a progressive pathological development in MASH, is primarily driven by hepatic stellate cells (HSCs), whose activation is strongly influenced by the pro-inflammatory environment established by KCs [22,23]. Pro-inflammatory cytokines, particularly TGF-β released from activated KCs, act as potent stimuli for HSC activation [23]. These cytokines induce the transformation of HSCs into myofibroblast-like cells, the primary cellular source of excessive extracellular matrix (ECM) deposition [24]. Furthermore, ROS derived from KCs also directly contribute to HSC activation and ECM deposition [24]. The renin–angiotensin system (RAS) further exacerbates fibrogenesis in MASH. Angiotensin II (AngII), generated locally by activated HSCs, directly stimulates HSC activation and collagen production. Simultaneously, AngII induces the release of profibrotic factors such as TGF-β1, which in turn promotes further HSC activation and AngII synthesis, thereby establishing a self-amplifying loop that perpetuates fibrogenesis [25,26]. Together, these mechanisms establish a vicious cycle of sustained ECM accumulation, culminating in irreversible architectural disruption and liver dysfunction.

**Figure 1:**
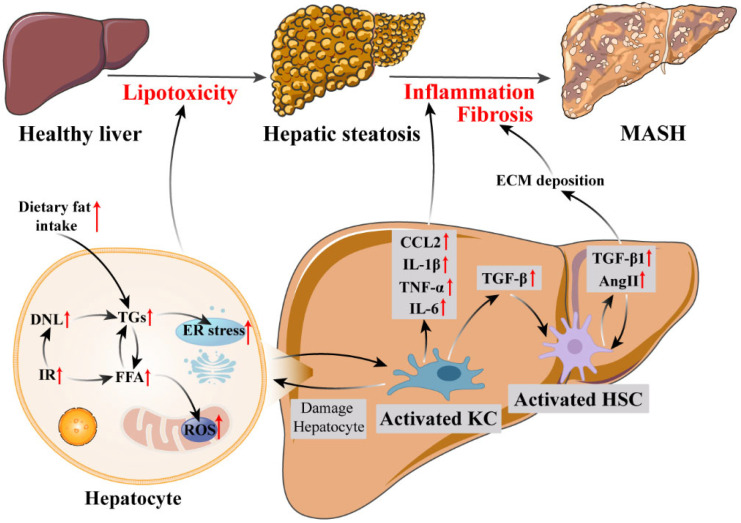
The schematic diagram of MASH pathological mechanisms. Increased dietary fat intake, elevated DNL, and IR lead to increased FFA influx, resulting in TG accumulation in hepatocytes and causing oxidative stress and ER stress, which subsequently induces the release of lipotoxic and injury signals. Injured hepatocytes activate KCs and release cytokines (TNF-α, IL-1β, IL-6) and chemokines (CCL2). These inflammatory mediators amplify hepatocyte injury and oxidative stress and drive HSC activation. Activated HSCs are further stimulated by inflammatory mediators, AngII, TGF-β, and TGF-β1, leading to excessive ECM deposition and liver fibrosis. DNL, de novo lipogenesis; IR, insulin resistance; FFA, free fatty acid; TG, triglyceride; ER stress, endoplasmic reticulum stress; KC, Kupffer cell; HSC, hepatic stellate cell; ECM, extracellular matrix. Red arrows indicate upregulation.

In summary, MASH pathogenesis is a sequential process initiated by disordered lipid metabolism and IR, leading to hepatic steatosis and subsequent lipotoxicity. Hepatocyte injury resulting from lipotoxicity triggers KC activation and the release of pro-inflammatory mediators, establishing chronic hepatic inflammation. This inflammatory environment, orchestrated by KCs and reinforced by signals from injured hepatocytes, subsequently drives HSC activation, culminating in ECM deposition and the development of liver fibrosis (Figure 1).

## 3. Transcriptomics

The transcriptome encompasses the entire collection of RNA transcripts within a specific tissue or cell, offering a snapshot of gene expression patterns [27]. Transcriptomic analysis, utilizing microarrays or RNA sequencing (RNA-seq), allows researchers to study gene function and structure at a global level, uncovering molecular mechanisms underlying disease pathogenesis [27]. In MASH, transcriptomic studies have focused on gene expression in liver tissue and peripheral blood, aiming to identify potential biomarkers and understand the intricate interplay of genes driving disease progression.

### 3.1. Gene Expression and Transcriptome Profiling Analysis

The application of transcriptomics has greatly improved our understanding of the pathogenesis of MASH. Early bulk RNA sequencing studies provided fundamental insights, revealing widespread transcriptional dysregulation. These studies highlighted the sustained upregulation of genes that drive adipogenesis, such as *SREBP1c* and *ACSL4* [28,29], and profibrotic and inflammation-driving genes, such as *IL-32* and *TNF-α* [30], as well as downregulation of genes involved in glucose, fatty acid and amino acid metabolism and mitochondrial function, such as *CRLS1* [31]. This dysregulation is reflected in the activation of inflammatory signaling pathways and fibrosis progression, such as Notch, TGF-β, NF-κB, and PI3K-AKT [32,33], and changes in the expression of ECM remodeling genes, such as *COLs*, *IGFBP7*, *PDGFRA*, *THBS2*, *EFEMP1*, and *DPT* [34,35].

Particularly, *ACSL4* not only promotes hepatic lipogenesis, lipid accumulation, and inflammatory progression, but also plays a dual role in autophagy and ferroptosis. *ACSL4* upregulation promotes lipid accumulation and inflammation, leading to ER stress and mitochondrial dysfunction, which in turn leads to *CRLS1* downregulation and *ATF3* upregulation [31,36]. *ATF3* upregulation further exacerbates ER stress, mitochondrial dysfunction, and inflammation [36]. Inhibition of *ACSL4* can promote mitochondrial respiration and fatty acid β-oxidation by upregulating *PPARα*, thereby reducing lipid accumulation [37]. *PPARα*, as a key regulator of lipid metabolism, can also affect the expression of *DPT* through the *Klf6/TGFβ1* pathway, which reveals the potential link between lipid metabolism and the development of MASH fibrosis [38]. Many bulk-seq studies have demonstrated the vicious cycle between MASH lipid metabolism, inflammation, and fibrosis [30,33]. However, due to the inherent heterogeneity of liver tissue, bulk-seq cannot analyze cell type-specific contributions, nor can it identify and study the role and regulatory mechanisms of these differential genes in different liver-type cells.

The advent of single-cell RNA sequencing (scRNA-seq) and spatial transcriptomics has overcome this limitation, providing crucial insights into the cellular heterogeneity of MASH. Studies employing scRNA-seq and spatial transcriptomics on human liver biopsies and mouse models have revealed critical roles for both HSCs and immune cells [39,40,41]. Analysis of human MASH livers demonstrated a significant increase in senescent HSCs, which were highly positively correlated with profibrotic gene expression. These senescent HSCs, derived from activated HSCs, displayed heterogeneous *PLAUR* expression [39]. Notably, a subset of senescent HSCs exhibited high *PLAUR* expression, particularly in later stages of MASH, and showed spatial association with infiltrating *CCR2*+*Ly6Chi* bone marrow-derived monocytes [39]. This suggests a dynamic interplay between senescent HSCs and the inflammatory microenvironment, potentially contributing to fibrosis progression. Five genes (*Sema6a*, *Mrc1*, *Stab2*, *Ptprb*, *Slc9a9*) were specifically upregulated in senescent HSCs, providing potential markers for this cell population [39].

In mouse models, studies identified dysregulated genes within macrophage populations. Macrophage-specific *NCF1* upregulation was found to exacerbate MASH through *TLR4*-mediated ferroptosis [40], while disruption of pericyte signaling modules in HSCs promoted HSC activation and fibrosis [41]. Triggering receptor expressed on myeloid cells 2 (*TREM2*) plays a pivotal role in shaping the function of a specialized subset of disease-associated macrophages. These *TREM2*+ macrophages are particularly important in maintaining tissue homeostasis and resolving inflammation, especially in conditions like MASH [42]. Their crucial functions include efficient efferocytosis, and the clearance of apoptotic cells and cellular debris, thereby reducing inflammation and promoting tissue repair [43]. Furthermore, *TREM2*+ macrophages actively participate in lysosomal degradation and lipid metabolism, crucial for processing cellular waste and excess lipids accumulating in the liver during MASH [44,45]. This metabolic activity, coupled with their ability to modulate inflammatory responses and contribute to ECM remodeling, positions *TREM2*+ macrophages as key regulators of liver health.

Beyond characterizing individual cell types, scRNA-seq has illuminated dynamic intercellular interactions and revealed remarkable cellular plasticity in MASH. While previous studies often considered HSCs and immune cells as distinct entities, emerging evidence emphasizes their complex interplay. For example, activated HSCs recruit immune cells by secreting numerous chemokines, including *MCP-1*, *IL-8*, *RANTES, CCR5*, and *SDF-1/CXCL12* [42,46,47]. Reciprocally, immune cells modulate HSC behavior. Macrophages, a key source of *TGF-β*, *PDGF*, *TNF*, and *IL-1β*, drive HSC activation and survival [42]. Lymphocytes, including B and CD8+ T cells, release HSC-activating cytokines like *TNFα* and *IL-6* [48]. The intricate bidirectional communication between HSCs and immune cells underscores their close functional relationship in MASH pathogenesis [49]. This dynamic interplay is not limited to HSCs and immune cells; cellular plasticity, a hallmark of MASH, extends to other liver cell populations as well. A prime example of this plasticity is the hepatocyte-cholangiocyte transdifferentiation observed in MASH. A study utilizing cholangiocyte organoids (ICOs) and human liver biopsies, leveraging single-cell RNA sequencing, found that activation of the PI3K-AKT-mTOR signaling pathway promotes the transdifferentiation of cholangiocytes into hepatocyte-like cells [50]. Inhibiting this pathway reduced this transdifferentiation and attenuated liver inflammation and fibrosis in a mouse model of MASH. This finding highlights the previously underestimated mechanism of MASH progression and adds another layer of complexity to the already intricate interactions between various liver cell types.

### 3.2. MicroRNAs and Long Non-Coding RNAs

MicroRNAs (miRNAs) and long noncoding RNAs (lncRNAs) are involved in core pathological processes driving the progression of MASH, and several miRNAs have been identified as key regulators of hepatic lipid handling. *MiR-122* is highly enriched in the liver and is consistently downregulated in MASH, and its downregulation may promote lipogenesis and accumulation by upregulating genes involved in fatty acid synthesis (*FASN* and *SREBP1c*) [51,52,53]. In contrast, *miR-33* was specifically elevated in the CD-HFD-fed mouse model and increased hepatic fat accumulation and degeneration by reducing mitochondrial fatty acid oxidation and increasing lipogenesis [54]. Similarly, upregulation of *miR-484* and *miR-22* promoted hepatic lipid accumulation and increased apoptosis, which may be achieved by *miR-484* through downregulating *Sorbs2*, a protein that may be involved in mitochondrial β-oxidation [55,56].

MASH inflammatory and fibrotic processes are also significantly affected by miRNAs. Both *miR-34a* and *miR-192* are upregulated in MASH and promote fibrosis [57,58,59], although through different mechanisms: *miR-34a* by inhibiting the anti-fibrotic protein *SIRT1* [60], whereas *miR-192* may act by upregulating *SREBF1* [61]. Similarly, *miR-21* promotes inflammation and fibrosis by targeting *HMGCR* and affecting *PPARα* [62,63]. Differently, *miR-145a-5p* downregulation promotes MASH progression by upregulating the nuclear receptor *Nr4a2* involved in inflammation [64], while *miR-552-3p* exhibits a protective effect by inhibiting HSC activation and reducing the expression of inflammatory and fibrotic genes [65]. Interestingly, *miR-665-3p* appears to exacerbate MASH inflammation and oxidative stress by inhibiting the *FNDC5/AMPKα* pathway [66]. Exosomal miRNAs further highlight the role of intercellular communication in MASH pathogenesis. For instance, M2 macrophage-derived exosomes effectively inhibited HSC activation by downregulating *CAMSAP1* in HSCs through the delivery of *miR-411-5p*, which represents a novel mechanism by which M2 macrophages may alleviate MASH-related fibrosis through exosome-mediated signaling [67]. These findings underscore the complex and multifaceted roles of miRNAs, acting both within cells and mediating intercellular communication, in regulating inflammation and fibrosis in MASH.

LncRNAs also contribute to MASH pathogenesis. LncRNAs such as *lncRNA NEAT1* and *lncRNA MALAT1* promote steatosis through various mechanisms, including upregulation of *AQP7* [68], *FAS*, and *ACC* [69] expression and activation of *miR-139-5p/c-Jun/SREBP1c* [70] and *miR-206/ARNT/PPARα* pathways [71], respectively. Similarly, *lncRNA H19* can further promote lipid accumulation by stimulating PPAR signaling and interacting with *PTBP1* [72]. This highlights the complex regulatory network controlling hepatic lipid metabolism in MASH. *LncRNA GAS5* and *lncRNA MEG3* are typical examples of lncRNA and miRNA interactions regulating inflammation and fibrosis. *LncRNA MEG3* alleviates inflammation through regulating the *EZH2/SIRT6* pathway [73]. It also competes with *LRP6* for *miR-21* binding, thereby reducing the inhibitory effect of *miR-21* on *LRP6* expression, thus activating the mTOR pathway and reducing lipid accumulation in hepatocytes [74,75]. *LncRNA GAS5* exacerbates fibrosis by interacting with both *PTEN*/PI3K/Akt/*miR-23a* [76] and *miR-29a-3p*/NOTCH2 axis [77]. These findings reveal the complex roles of miRNAs and lncRNAs in MASH pathogenesis, demonstrating the intricate regulatory network governing MASH development.

### 3.3. Diagnostic Model Based on Transcriptomics

Transcriptomic data have led to the development of several promising diagnostic models for MASH. These models, often incorporating miRNAs, lncRNAs, or mRNAs, show potential for improved diagnostic accuracy compared to existing non-invasive methods and could offer a viable alternative to liver biopsy (Table 1). Recently, widely used models like FibroScan-AST [78], Fibrosis-4 score (FIB-4) [79], and Aspartate Aminotransferase-to-Platelet Ratio Index (APRI) [80], originally developed for NAFLD, have been validated in MASLD patients. However, their performance in diagnosing MASH, identifying high-risk MASH, and distinguishing fibrosis stages remains suboptimal. The accuracy of most of these models falls below 0.8, often fluctuating considerably within the range of 0.6 to 0.85 [81,82]. This highlights the need for improved non-invasive diagnostic tools, and transcriptome-based models offer a potential solution with their higher diagnostic efficiency.

*MiR-122* has been identified as a potential diagnostic biomarker by multiple studies, and it can accurately predict the presence of MASH, but its diagnostic efficiency is significantly poor when distinguishing pure lipid degeneration [58,83,84,85]. *MiR-34a*, consistently linked to increased fibrosis in MASH, has also been investigated as a potential biomarker [86,88]. However, its utility as a standalone biomarker may be limited due to its involvement in other liver diseases as well [83,99]. The limitations of single biomarkers have prompted research into multi-miRNA diagnostic panels for MASH. Groups like *miR-122*, *miR-192*, and *miR-375* show promise in differentiating early and late-stage MASH. This suggests that miRNA combinations may offer increased diagnostic accuracy. However, present multi-miRNA panels do not outperform existing scoring systems like FIB-4 and NFS [58]. Furthermore, they are unable to reliably distinguish simple steatosis from MASH.

*TREM2* has emerged as a promising biomarker for MASH/MASLD. Studies indicate it can effectively differentiate patients with cirrhosis from those with simple steatosis. It can also distinguish MASH patients from healthy individuals. *TREM2* boasts high diagnostic accuracy rates of 0.907 and 0.883, respectively, for these distinctions, outperforming traditional liver disease parameters [44]. Other studies have found that the expression of *THBS2* in MASH is significantly higher than that in simple steatosis and increases synchronously with the stage of fibrosis. A predictive model based on TSP-2, encoded by *THBS2*, has shown promise in MASH/MASLD prognosis. This model predicts MASH and advanced fibrosis with AUROCs of 0.776 and 0.856, respectively [35]. This performance is comparable to established methods like the FIB-4 index, serum HA levels, and NFS in diagnosing advanced fibrosis. Therefore, TSP-2 may be useful for stratifying MASLD patients based on complication risk. In addition to using RNA as an independent biomarker, researchers are also exploring its combination with other clinical data to develop more effective diagnostic models. One study combined the cytokine *IL-31* with traditional liver enzymes to improve the diagnostic performance of MASLD/MASH compared with the traditional ALT-AST model [30]. Likewise, the combination of *TGFB2/TGFB2-OT1* + FIB-4 (AUC = 0.891) or *TGFB2/TGFB2-OT1* + FibroScan (AUC = 0.892) was able to robustly discriminate patients with F3-4 from patients with F0-2 [76]. These studies demonstrate the benefit of combining RNA biomarkers with existing clinical parameters.

Although many studies have shown that lncRNAs are significantly dysregulated in MASH patients/mice, studies using lncRNAs to construct MASH diagnostic models are rare. For example, *HSPA5*, a chaperone that controls fat metabolism in the endoplasmic reticulum, is highly expressed in MASH and can precipitate mRNAs such as *EGFR* which promotes fibrosis, and *TGFβ1* that promotes inflammation [100]. *Kcnq1ot1* and *Rmst* are downregulated in CCL4-induced MASH in mice and may serve as biomarkers to distinguish the fibrosis stage of MASH, but none of these lncRNAs have been shown to be able to diagnose MASH or distinguish the fibrosis stage [101]. In the rare studies on the use of lncRNA to construct a diagnostic model for MASLD, *lnc PVT1* and *HCG18* can well distinguish MASLD patients from normal controls, with diagnostic efficiencies of 0.895 and 0.934, respectively [87,89]. However, the diagnostic model constructed by Plasma *lncRNA LeXis* and *lncRNA TGFB2/TGFB2-OT1* to distinguish MASH from normal controls is very worrying, with diagnostic efficiencies of 0.743 and 0.797, respectively [76,90]. So far, the most efficient lncRNA for distinguishing MASH constructed using lncRNA is *lnc SPARCL1-1:2*, with a diagnostic efficiency of 0.87, and it can also distinguish simple steatosis from MASH and MASLD from MASH, with diagnostic efficiencies of 0.79 and 0.974, respectively [91]. It is the lncRNA with the highest diagnostic efficiency and the widest range of applications among all lncRNAs.

Machine learning (ML) algorithms, particularly supervised methods analyzing multi-omics datasets including transcriptomics, are increasingly utilized to develop more objective and reliable diagnostic models for MASH, surpassing traditional approaches by leveraging complex data patterns to reveal biomarkers and improve diagnostic accuracy [10]. Currently, several studies have used ML to develop diagnostic models of MASH and identify key genes associated with the MASH/MASLD. Several key genes were related to cell mitophagy, including *MRAS* (AUC: 0.885), *RAB7B* (AUC: 0.884), and *RETREG1* (AUC: 0.7) [82,92,93]. Another set of genes, possibly linked to cell exocytosis, comprises *TREM2* (AUC: 0.955), *TIMD4* (AUC: 0.966), *STAB1* (AUC: 0.489), *C1QC* (AUC: 0.568), and *DYNLT1* (AUC: 0.716) [94]. Oxidative stress may be reflected in the expression of *NDUFA4* (AUC: 0.935), *TFAM* (AUC: 0.909), and *CDKN1B* (AUC: 0.911) [82]. Cuproptosis-related genes include *NFE2L2*, *DLD,* and *POLD1* (AUC: 0.97) [95]. Furthermore, a set of eight ferroptosis-related genes (FeRS, AUC: 0.9) [92,96] has been identified, as well as key genes in immune cell populations, including *ZFP36L2* (AUC: 0.721) and *PHLDA1* (AUC: 0.753) [97,98,102]. Furthermore, a recent study employing a machine learning-based histopathology approach developed a 5-gene signature (*JAG1*, *VIM*, *VWF*, *PDGFRA*, and *CLSTN1*) that demonstrates strong predictive value for severe MASH (F3–F4 fibrosis) and clinical outcomes, highlighting the power of ML to integrate complex data and improve diagnostic and prognostic accuracy in MASH [32]. These advanced ML-based diagnostic models offer a more objective and reliable approach to MASH diagnosis and provide a pathway for non-invasive and personalized diagnostic tools.

## 4. Proteomics

Proteomics systematically studies the structure, function, and dynamic interactions of proteins, which execute essential cellular processes ranging from catalysis to signal transduction [103]. Utilizing advanced techniques such as mass spectrometry and bioinformatics enables comprehensive profiling of protein expression, post-translational modifications, and molecular networks, offering critical insights into disease mechanisms for biomarker discovery and therapeutic development [103]. Proteomic studies have provided valuable insights into the molecular mechanisms underlying MASH, identifying differentially expressed proteins and altered pathways associated with disease progression. These studies can be roughly divided into clinical findings based on serum/plasma proteomics and MASH pathogenesis studies through liver proteomics.

### 4.1. Protein Expression and Protein Profiling Analysis

Studies focusing on serum and plasma have identified several proteins with altered levels in MASH patients. Many inflammatory proteins are significantly increased in MASH patients, such as soluble CDCP1 (sCDCP1), IL-18, IL-6, ADA, EN-RAGE, Flt3L, and ST1A1 [104,105]. In particular, serum sCDCP1 levels were significantly elevated in NASH patients (223.01 pg/mL [125.47–352.59 pg/mL]) compared with NAFL (72.84 pg/mL [48.54–102.60 pg/mL]) and normal liver individuals (52.31 pg/mL [25.81–74.56 pg/mL]) [105]. Moreover, a large number of studies indicate that C-reactive protein (CRP) is significantly elevated in the serum of MASH patients [106]. However, it is crucial to note that all of these inflammatory protein elevations are not specific to MASH. These markers are also commonly elevated in other chronic liver diseases, such as hepatitis B and C, indicating they reflect a general hepatic inflammatory response rather than a MASH-specific signature [107,108]. And as the progression of fibrosis/cirrhosis, the levels of these inflammatory markers often increase further, which may be related to the increase in systemic inflammation [109]. Therefore, the identification of truly MASH-specific inflammatory protein biomarkers remains an ongoing challenge, highlighting the complex and heterogeneous nature of inflammatory responses in MASH.

In addition to inflammatory markers, serum and plasma proteomic studies have identified proteins associated with MASH-related fibrosis. During MASH progression, the protein profile secreted by HSC changes, with upregulated expression of ECM synthesis and remodeling proteins like ADAMTSL2, COL6A1, and COL6A2 [110]. ADAMTSL2 shows promise as a biomarker for MASH and fibrosis, with serum levels correlating with fibrosis severity [111]. However, the role of ADAMTSL2 in other liver diseases remains unclear, requiring further validation. Although some studies have shown that proteins such as THBS2, IGFBP7, and IGFPB2 are differentially expressed between MASLD and cirrhosis [112,113]. This suggests their potential role in the progression of fibrosis, but alterations in the GH/IGF axis and IGF-binding proteins are common in all chronic liver diseases [114]. This commonality limits their MASH-specific diagnostic value. Furthermore, proteins related to inflammation and immune response, including LGALS3BP, NRP1, ALPL, and ALDOB, are also upregulated in MASH fibrosis [115,116]. However, these fibrosis-associated protein biomarkers are also not specific to MASH-related fibrosis, and future research should prioritize the identification of biomarkers specific to MASH fibrosis [117].

Hepatic proteomics provides critical insights into key protein and pathway dysregulation in MASH, revealing disease pathogenesis. Comprehensive proteomic profiling of human and murine MASH livers identified 254 and 1917 differentially expressed proteins, respectively [118]. These proteins were predominantly enriched in pathways associated with PPAR signaling, AMPK signaling, insulin signaling, and fatty acid metabolism [118]. Cross-species analysis revealed 14 consistently dysregulated proteins, including lipid metabolism regulators such as Rbbp4, Tceal, and ILF2 [118]. Proteomic methods revealed that ATG3 expression was elevated in the liver of MASLD patients and mouse models, and its inhibition improved steatosis induced by p63 and diet [119]. Mechanistically, ATG3 reduced mitochondrial function by increasing JNK1 and reducing SIRT1 and CPT1a, thereby increasing lipid accumulation [119]. Proteomic profiling further identifies glycogen synthase kinase-3β (GSK3β) as a central kinase hub in MASH liver sinusoidal endothelial cells (LSECs), driving lipotoxicity-induced proinflammatory responses [120]. GSK3β activation in LSECs promotes myeloid cell transendothelial migration by upregulating chemokines CXCL2 and adhesion molecules ICAM-1, thereby mediating critical inflammatory processes [120]. Despite these advances, further studies are required to fully elucidate the mechanistic roles and functional consequences of these proteomically identified proteins and pathways in MASH pathogenesis.

### 4.2. Diagnostic Model Based on Proteomics

Proteomics offers a promising approach for improving the diagnosis of MASH by identifying novel protein biomarkers of MASH in various biological samples. Proteomic analysis, using techniques like mass spectrometry, provides comprehensive protein profiles in samples such as plasma, urine, and feces. These profiles, under similar physiological conditions, can reflect underlying metabolic and disease states within the liver, offering valuable insights for developing more accurate and specific diagnostic tools [121]. Traditional diagnostic models for MASH, such as NFS and FIB-4, rely on standard liver enzymes like AST and ALT but often lack sufficient accuracy and specificity for MASH, particularly in distinguishing it from other causes of liver disease. However, incorporating protein biomarkers identified through proteomic studies can enhance diagnostic performance. For example, while commonly used models incorporating enzymes like ALT and AST lack specificity for MASH, incorporating other proteins like PLIN2 has demonstrated improved performance and specificity compared to standard models like FIB-4 [122]. Similarly, sTREM2 and TSP-2 have shown promise in differentiating cirrhosis and simple steatosis, and in distinguishing MASH patients from healthy individuals [111,123,124,125]. These findings suggest that integrating proteomic biomarkers holds the potential for developing more accurate and specific diagnostic tools for MASH (Table 2).

Recognizing the complex and multifaceted nature of MASH, researchers have explored multi-protein diagnostic models. Sanyal et al. developed a four-protein model for diagnosing various MASLD components, highlighting the disease’s heterogeneity with only two analytes (PTGR1 and ADAMTSL2) shared across models [126]. ADAMTSL2 has emerged as a particularly strong biomarker candidate, demonstrating the ability to differentiate fibrosis stages in MASH and outperforming traditional scoring systems [127]. Multi-protein panels incorporating ADAMTSL2 and other proteins have also shown efficacy in distinguishing different stages of MASH fibrosis [127].

Expanding beyond serum and plasma, studies utilizing fecal and urine proteomics have demonstrated remarkably high diagnostic accuracy. The fecal metaproteomic analysis identified a seven-metaprotein model with near-perfect accuracy in distinguishing MASH from healthy individuals and even hepatocellular carcinoma [130]. Urine proteomics has also shown excellent performance in diagnosing MASLD presence and distinguishing fibrosis stages [128]. These findings underscore the potential of proteomics, particularly when leveraging multiple biomarkers and exploring alternative biological samples like urine and feces, to develop highly accurate and non-invasive diagnostic tools for MASH. However, further research is needed to validate these findings in larger, independent cohorts and translate promising results into clinically applicable diagnostic tests.

The study by Sourianarayanane et al. further highlights the complexity of biomarker discovery in MASH, namely the discordance between plasma and liver protein expression, suggesting that plasma biomarkers alone may not fully reflect liver pathology. They also found that incorporating liver tissue data can improve the accuracy of plasma-based models [129]. Taken together, these studies underscore the need for integrated approaches, combining multiple biomarkers and potentially incorporating liver-specific information, to develop robust and clinically relevant diagnostic models for MASH.

## 5. Metabolomics and Lipidomics

Lipidomics and metabolomics are two powerful omics technologies that provide unique insights into the complexity of MASLD and its more severe counterpart, MASH. Metabolomics, the comprehensive study of all small molecule metabolites in a biological system, provides a snapshot of metabolic status and reveals functional changes associated with the disease [131]. As the omics closest to the phenotype, metabolomics provides unique insights into the dysregulated pathways of MASH/MASLD. Lipidomics, the comprehensive study of lipids in a biological system, provides a detailed view of lipid composition, distribution, and function, providing valuable insights into the complex roles of lipids in health and disease [132]. In recent years, several advanced technologies have emerged in these fields: single cells, spatial metabolisms, and lipidomics, such as SpaceM, matrix-assisted laser desorption/ionization mass spectrometry imaging (MALDI-MSI), and spatial system technologies, which are able to study the spatial distribution of metabolites and lipids within tissues or cells, revealing how metabolites such as lipids interact with different cell types and microenvironments [133,134,135]. The development of these technologies has opened up new possibilities for a deeper understanding of the role of metabolites such as lipids in health and disease.

### 5.1. The Role of Lipid and Metabolite Remodeling in MASH/MASLD Pathogenesis

Lipid metabolism alterations are central to the pathogenesis of MASH/MASLD. Within this context, sphingolipids, a class of bioactive lipids with diverse signaling functions, emerged as critical players in disease development. Sphingolipids are involved in a variety of cellular processes, including inflammation, apoptosis, and IR, all of which are dysregulated in MASH/MASLD. A consistent finding across multiple studies is the accumulation of ceramides, particularly long-chain ceramides such as C16:0 and C22:0, in the livers of MASH/MASLD models [136,137,138]. This ceramide accumulation may be driven by increased ceramide synthase activity, decreased ceramide degradation, and/or enhanced sphingomyelinase activity. Additionally, glycosylceramides, including HexCer and Hex2Cer, are also elevated in HFD models [137]. Sphingomyelin (SM), the most abundant sphingolipid, exhibits altered metabolism in MASH/MASLD, characterized by changes in acyl chain distribution and increased levels of SM C16, SM C18, and other SM [136,137,139]. Collectively, these findings point to a profound dysregulation of sphingolipid metabolism in MASH/MASLD.

Except for sphingolipids, a complex interplay of other lipid alterations contributes to the MASH/MASLD lipidome. Reduced hepatic phosphatidylcholine (PC) levels are a consistent observation across multiple studies, including both MCD and CDAHFD-induced MASH mouse models and pediatric NAFLD [140,141]. In the CDAHFD model, which more closely resembles human NASH pathology, this PC reduction is accompanied by a downregulation of *Pcyt1a*, a key gene in PC synthesis [141]. A shift in PC species distribution is observed in children with NAFLD, with increased shorter, saturated PCs and decreased longer, polyunsaturated PCs, a pattern also observed in adults with hepatic steatosis [140]. These children also exhibited increased saturated lysophosphatidyl choline (LPC), including LPC (18:0), and total LPC, as well as decreased phosphatidylglycerols and gangliosides, compared to obese children. Ooi et al. corroborated these findings, showing that PCs were lower in livers with more severe steatosis, and that specific PCs, lysophosphatidylethanolamines (LPEs), phosphatidylinositols (PIs), and lysophosphatidylinositols (LPIs) exhibited substantial changes [138]. They also found increases in free cholesterol and variable changes in cholesterol esters, substantial increases in total TG and diacylglycerol (DG), as well as increases in most TG and DG species. Acylcarnitines, involved in mitochondrial fatty acid oxidation, were also reduced in the CDAHFD model and in older female mice fed an HFD [137,141]. While these studies suggest a role for PC, acylcarnitine, and other lipid classes in MASH/MASLD, the complex interplay of these alterations and the precise mechanisms by which they drive disease progression remain largely unknown. These findings underscore the need for further research to fully elucidate the intricate lipid dynamics in MASH/MASLD.

In addition to lipids, alterations in bile acid (BA) metabolism are also associated with the progression and severity of MASH/MASLD. BAs, synthesized from cholesterol in the liver, are essential for lipid digestion and absorption. Serum metabolomics of MASLD patients revealed significant changes in the levels of several BAs, including glycocholic acid (GCA), glycochenodeoxycholic acid (GCDCA), glycodeoxycholic acid (GDCA), glyco lithocholic acid (GLCA), lithocholic acid (LCA), tauro lithocholic acid (TLCA), and tauroursodeoxycholic acid (TUDCA), in NAFLD patients. Notably, the enhanced liver fibrosis score (a marker of liver fibrosis) showed a high positive correlation with total BAs, cholic acid (CA), primary BAs, chenodeoxycholic acid (CDCA), taurine-conjugated BAs, glycine-conjugated BAs, and non-12-α-OH BAs [142]. Similarly, fecal metabolomics of MASH patients found that the levels of deoxycholic acid (DCA) and its microbial metabolites (glycodeoxycholic acid, 7-ketodeoxycholic acid, and dehydrocholic acid) increased with increasing disease activity and fibrosis stage [143]. Besides, serum levels of BAs (such as taurocholic acid, etc.), which are significantly elevated in MASH patients, are positively correlated with fecal metabolites N,N,N-trimethyl-5-aminovaleric acid that predict liver cirrhosis [144]. These studies mutually highlight that dysregulation of BA metabolism is a key feature of MASH/MASLD, closely associated with the progression of MASH. However, BA dysregulation is a common feature of viral hepatitis and advanced chronic liver disease and is not unique to MASLD/MASH [145]. Further studies are needed to determine whether BA dysregulation mechanisms differ across liver diseases.

Amino acids, as components of proteins, play an important role in energy metabolism, immune function, and cell signaling, and their metabolism is also altered in MASH/MASLD patients [146,147]. Alterations in amino acid metabolism, such as increased branched-chain and aromatic amino acids, along with a decrease in amino acids involved in glutathione metabolism, may be indicative of insulin resistance, oxidative stress, and inflammation [148]. One untargeted metabolomics study showed that elevated levels of alanine, histidine, and tyrosine are related to the severity of NAFLD [149]. In male subjects, serum concentrations of valine and aspartic acid are positively correlated with NAFLD progression, while in female subjects, threonine levels are negatively correlated with NAFLD progression [149]. Interestingly, hepatectomized MASH mice exhibit further amino acid metabolic alterations compared to healthy controls, with dysregulation in phenylalanine, tyrosine, and tryptophan biosynthesis as well as arginine/proline pathways, all of which may contribute to the complex process of liver regeneration. In addition, levels of organic acids (intermediates of various metabolic pathways) were also altered, such as decreased citrate and α-ketoglutarate. These changes, often observed in MASH/MASLD, may reflect mitochondrial dysfunction and impaired tricarboxylic acid (TCA) cycle activity, contributing to the metabolic dysregulation characteristic of the disease [150]. Reduced TCA cycle activity can lead to decreased adenosine triphosphate production and increased oxidative stress, further exacerbating liver damage in MASH.

Single-cell and spatially resolved omics technologies are revolutionizing our understanding of MASH by revealing its complex metabolic landscape. Traditional bulk tissue analyses mask crucial cellular and spatial metabolic heterogeneity, which spatial metabolomics overcomes by utilizing techniques like MALDI-MSI and desorption electrospray ionization mass spectrometry imaging (DESI-MSI) [134,151]. These methods enable spatial mapping of metabolites within tissue sections, providing insights into compartmentalization and cellular interplay. For instance, MALDI-MSI studies by Stopka et al. revealed spatially heterogeneous metabolic reprogramming in HFD-fed mice, characterized by a unique oxidative stress signature with localized PPP activation but systemic NADPH depletion, highlighting compartmentalized metabolic dysfunction in MASH [135]. Furthermore, the SpaceM method, integrating metabolic profiling, morphometrics, and fluorescence intensity, has identified distinct hepatocyte metabolic states (steady, intermediate, steatotic) correlating with lipid droplet levels and revealed that inflammatory stimuli (IL-17A) induce a shift towards a steatotic state characterized by increased ceramide accumulation, highlighting inflammation’s role in exacerbating metabolic reprogramming and lipid accumulation in MASH [133]. These spatially resolved techniques highlight the importance of considering metabolic heterogeneity and spatial context to fully understand the complex metabolic dysregulation that drives MASH.

### 5.2. Diagnostic Models Based on Metabolomics and Lipidomics

Lipidomics and metabolomics, as the most phenotypical omics technologies, provide a unique perspective for developing non-invasive diagnostic models for complex metabolic diseases such as MASH. Many studies have explored the potential of these technologies in building diagnostic models for MASH and related liver diseases (Table 3). The metabolomics-advanced steatohepatitis fibrosis (MASEF) score, integrating 12 lipids, BMI, AST, and ALT, could identify high-risk MASH patients and achieve an AUROC of 0.79 in the validation cohort, showing its potential to replace liver stiffness measurement and become part of the existing diagnostic algorithm [152]. The OWLiver Panel model, integrating OWLiver score for diabetes mellitus 2 (OWLiver DM2) and MASEF, also showed good ability to distinguish high-risk MASH patients with an AUROC of 0.788, as well as to differentiate MASH patients from healthy individuals and isolated steatosis from MASH, with accuracies of 0.861 and 0.77, respectively [153]. In addition, a MASLD diagnostic model developed based on the plasma eicosanoid profile of 382 severely or morbidly obese patients from six European centers had an AUROC of up to 0.999 [154]. This suggests that eicosanoids may be potential biomarkers for MASLD and may play a role in the diagnosis of MASH, but this needs to be validated in the MASH population.

Other studies have explored the potential of specific lipid species as biomarkers for MASLD/MASH. For example, research has shown that specific lipid species exhibit distinct trends during MASLD progression and can differentiate between simple steatosis, MASH, and non-steatotic livers. These lipids include certain SMs, particularly SM 43:3;2 and SM 43:1;2 (containing n24:2 and n24:0 fatty acids), and several glycerophospholipids containing C22:6 fatty acids [162]. The study by Jung et al. developed a plasma lipid-based prediction model for MASLD and MASH that performed well in both obese and non-obese individuals, highlighting the value of circulating lipid profiles in disease diagnosis [155]. In addition, Z. H. Wang et al. found several plasma lipid species that were significantly altered in MASH patients, including PC (14:0/18:2), phosphatidyl ethanolamine (18:0/22:5), and PC (26:1/11:0) as well as specific plasmalogens [163]. Urinary EVs have also been shown to be a potential source of non-invasive biomarkers for MASH, with certain lipid molecules contained therein (e.g., LPC (22:6/0:0), FFA (18:0), FFA (18:1), and PI (16:0/18:1)) being effective in distinguishing MASH from simple steatosis patients [156]. These findings highlight the potential of lipidomics-based diagnostic models in MASH/MASLD.

In addition to lipids, some amino acids can also be used to diagnose MASH, and other MASH-related biomarkers can optimize the diagnostic model to improve the diagnostic efficiency of the model. The MASH ClinLipMet score was developed by targeted mass spectrometry analysis of the blood of 318 subjects who underwent liver biopsy for suspected MASH [158]. The score achieved an AUROC of 0.866 for distinguishing MASH, integrating indicators such as glutamate, isoleucine, glycine, LPC 16:0, phosphatidylethanolamine 40:6, AST, fasting insulin, and PNPLA3 genotype. However, its performance in distinguishing MASLD was less impressive, with an accuracy of 0.77, sensitivity of 0.86, specificity of 0.35, PPV of 0.85, and NPV of 0.36. Despite these limitations, the score still performs significantly better than those based solely on clinical or metabolic profiles. Similarly, another study used fecal microbiome and metabolomic data to develop a diagnostic model that was able to distinguish non-cirrhotic MASH patients from healthy controls with high accuracy [159]. These studies all emphasize that combining metabolomics with other MASH-related biomarker data can significantly improve the diagnostic performance of the model.

The application of ML methods provides a new way to integrate multi-omics and biochemical marker data and build a more accurate MASH diagnostic model. The MetaNASH score was constructed by combining targeted metabolomic analysis of plasma samples from 86 subjects with ML [161]. The score is based on three metabolites: glutamate, isocitrate, and aspartate, and achieves excellent diagnostic performance for MASH(AUROC:0.821). Leung et al. developed a machine-learning model that integrates baseline fecal microbiome, serum metabolomics, and clinical parameters [161]. The cohort of this prospective study included 2487 Chinese individuals, of whom 90 developed MASLD 4.6 years after baseline and 90 matched control individuals remained MASLD negative during follow-up. The model can predict the risk of individuals developing MASLD (including MASH) several years after baseline, and its accuracy even exceeds that of existing clinical prediction models. Similarly, another study developed a model for predicting MASH from lipidomic data of plasma samples from 12 MASH patients, 10 patients with simple steatosis, and 15 healthy controls combined with the XGBoost ML algorithm. The model integrates clinical indicators such as HOMA-IR, BMI, platelet count, and multiple lipid molecules, and the AUROC reaches 0.900 [157], further demonstrating the advantages of ML in integrating complex data and improving diagnostic efficiency.

## 6. Conclusions

**Figure 2:**
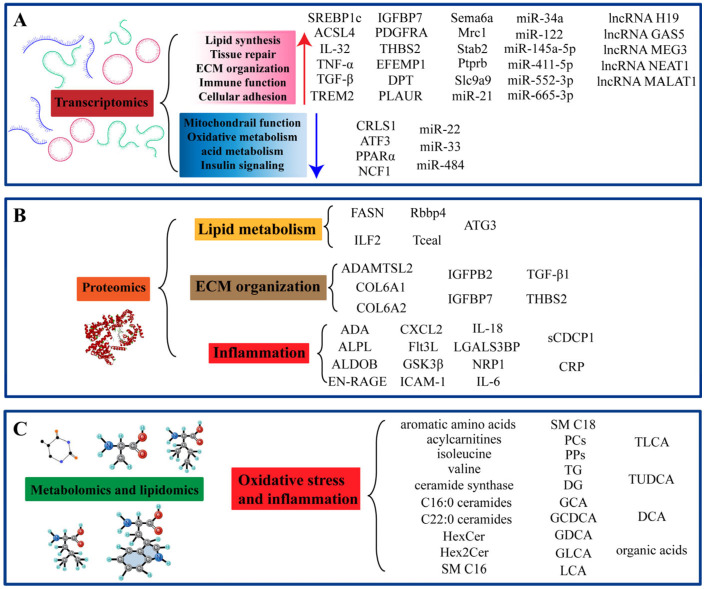
Potential biomarkers for MASH identified through multi-omics approaches. (**A**) Potential biomarkers for MASH discovered by transcriptomics. (**B**) Potential biomarkers for MASH discovered by proteomics. (**C**) Potential biomarkers for MASH discovered by lipidomics and metabolomics.

Multi-omics studies have revealed a complex and dysregulated molecular landscape driving MASH pathogenesis (Figure 2). Transcriptomic studies have demonstrated significant alterations in hepatic gene expression, notably the upregulation of genes promoting adipogenesis, lipid accumulation, and lipid metabolism—*SREBP1c*, *ACSL4*, *miR-33*, *lncRNA NEAT1*, and *lncRNA MALAT1*—and the downregulation of *CRLS1* and *miR-122* (Figure 2A). Pro-fibrotic and inflammation-driving genes, including *IL-32*, *TNF-α*, and *lncRNA GAS5*, are also upregulated. Single-cell RNA sequencing has identified some specific cell types and mechanisms, revealing macrophages exhibiting *NCF1* upregulation and TLR4-mediated ferroptosis, senescent HSCs marked by *Sema6a*, *Mrc1*, *Stab2*, *Ptprb*, *Slc9a9*, and cholangiocyte transdifferentiation into hepatocyte-like cells, driven by PI3K-AKT-mTOR pathway activation. Proteomic analyses have identified altered circulating levels of inflammatory and fibrosis-associated proteins in serum, including CDCP1, IL-18, IL-6, ADAMTSL2, COL6A1, and COL6A2 (Figure 2B). Hepatic proteomics has validated pathway-level dysregulation in PPAR, AMPK, and insulin signaling, and fatty acid metabolism, with specific proteins such as ACLY and FASN upregulated. Notably, transcription factors *Rbbp4*, *Tceal*, and *ILF2*, and their encoded protein products, display consistent expression changes across human and mouse models. Metabolomic and lipidomic investigations have revealed extensive lipid and metabolite remodeling. Key lipidomic alterations include the accumulation of sphingolipids—ceramides (specifically long-chain ceramides C16:0 and C22:0), glycosylceramides (HexCer and Hex2Cer), and specific SM species—alongside decreased hepatic PC levels and a shift in PC species distribution towards shorter, saturated forms (Figure 2C). Metabolomic studies have identified dysregulated BA metabolism, elevated branched-chain and aromatic amino acids, reduced amino acids related to glutathione metabolism, and decreased levels of organic acids, such as citrate and α-ketoglutarate, reflecting mitochondrial dysfunction. Spatially resolved metabolomics revealed compartmentalized oxidative stress signatures, predominantly localized to extravascular liver regions, indicative of spatial metabolic heterogeneity in MASH livers.

Omics data have also facilitated the development of non-invasive diagnostic models for MASH. Several MASH diagnostic models developed based on omics data exhibit excellent diagnostic performance, such as models incorporating *miR-34a* and multi-miRNA panels (*miR-122*, *miR-192*, *miR-375*). Machine learning-enhanced transcriptomic models have identified gene signatures with high diagnostic accuracy, including mitophagy-related genes (*MRAS*, *RAB7B*, *RETREG1*, etc.), oxidative stress-related genes (*NDUFA4*, *TFAM*, *CDKN1B*, etc.), and immune cell-related genes (*ZFP36L2*, *PHLDA1*). A histopathology-derived, machine learning-based 5-gene signature (*JAG1*, *VIM*, *VWF*, *PDGFRA*, and *CLSTN1*) also demonstrates strong predictive value for severe MASH. Proteomic-based diagnostic models include multi-protein panels incorporating ADAMTSL2 and models combining PLIN2 with traditional liver enzymes. Serum TREM2 and TSP-2 show promise for differentiating cirrhosis from simple steatosis and for distinguishing MASH patients from healthy individuals, respectively. Metabolomic and lipidomic-based models—such as the MASEF score, integrating PC, SM, and lysophosphatidylethanolamine, and the OWLiver Panel—and machine learning-enhanced metabolomic models (e.g., the MetaNASH score, based on glutamate, isocitrate, and aspartate) also exhibit excellent diagnostic performance, often outperforming conventional scores like FIB-4 and NFS. These advanced multi-omics diagnostic models offer the potential for non-invasive MASH diagnosis and risk stratification.

Despite the significant advancements in MASH multi-omics research, several key limitations necessitate further investigation. Single-cell RNA sequencing, while revealing senescent HSCs as a distinct population in MASH, also indicates their relatively small proportion (7–8%) among total HSCs, raising questions about the proportional contribution and functional significance of this limited senescent HSC population to overall MASH pathogenesis [39]. Similarly, while macrophages, particularly *TREM2+* macrophages, are implicated in MASH, the precise mechanisms underlying the induction and function of this unique macrophage population in resolving inflammation and maintaining tissue homeostasis in MASH remain fully characterized [42]. Proteomic and metabolomic studies, while identifying numerous differentially expressed proteins and metabolites, face inherent limitations in detecting lower abundance molecules, potentially obscuring the roles of crucial low-abundance regulatory factors in MASH initiation and progression [164,165]. Furthermore, while omics studies identify differential expression of proteins and metabolites, the precise mechanisms by which these altered protein and metabolite levels contribute to MASH pathology remain largely unknown. For diagnostic models, even those that show promise, further validation in large, diverse, and independent cohorts is needed to confirm their robustness, generalizability, and clinical utility in different MASH populations and to ensure that they outperform existing clinical prediction models in clinical practice [166]. Looking ahead, future research should prioritize mechanistic studies elucidating the functional roles of omics-identified biomarkers, rigorous validation of diagnostic models, enhanced detection technologies for low-abundance molecules, and refined spatial omics approaches to translate multi-omics insights into improved MASH management.

## Figures and Tables

**Table 1:** MASH/MASLD diagnostic model constructed by transcriptomics research.

RNA Transcript	Comparisons	Prediction Models	Sensitivity	Specificity	AUROC	Refs.
miRNA	(1) NAFLD—simple steatosis vs. NASH (2) NAFLD—simple steatosis vs. C	*MiR-122*	NA	NA	(1) 0.75 (2) 0.93	[83]
	NAFLD—simple steatosis vs. NASH	*MiR-122*	92%	85%	0.81	[84]
	NAFLD—simple steatosis vs. NASH	*MiR-122*	NA	NA	0.71	[58]
	NAFLD vs. C	*MiR-122-5p*, *miR-1290*, *miR-27b-3p*, and *miR-192-5p*	69.9%	83.7%	0.78	[85]
	NAFLD vs. C	*MiR-122*	NA	NA	0.82	[86]
	NAFLD vs. C	*MiR-20a-5p*	84%	84.6%	0.89	[87]
	NASH vs. C	*MiR-34a*	70.4%	68.7%	0.81	[88]
lncRNA	NASH with F3-4 vs. NASH with F0-2	*TGFB2/TGFB2-OT1*, and FIB-4 *TGFB2/TGFB2-OT1*, and FibroScan	NA	NA	0.891 0.892	[76]
	NAFLD vs. C	*LncRNA HCG18* and *miR-197-3p*	NA	NA	0.93	[89]
	NAFLD vs. NASH	*LncRNA LeXis*	54.3%	100%	0.74	[90]
	(1) NASH vs. C (2) NASH vs. simple steatosis (3) NAFLD vs. NASH	*LncRNA HSPD1* *lncRNA MMP14* *lncRNA ITGB1* *lncRNA SPARCL1-1:2* *miR-6881-5p*	(1) 88.8%, 86.3%, 90%, 83.8%, 90% (2) 85%, 65%, 70%, 70%, 72% (3) 89%, 78.2%, 74%, 90%, 70.9%	(1) 76.2%, 80%, 70%, 72.5%, 83.8% (2) 63%, 83%, 83%, 82%, 83% (3) 93%, 93%, 78%, 100%, 93%	(1) 0.897, 0.862, 0.858, 0.870, 0.891 (2) 0.712, 0.730, 0.695, 0.790, 0.823 (3) 0.939, 0.868, 0.825, 0.974, 0.916	[91]
mRNA	NAFLD vs. C	*AKR1B10*, *TYMS*, and *TREM2*	NA	NA	0.71	[92]
	MASH vs. C	*MRAS*, *RAB7B*, and *RETREG1*	NA	NA	0.93	[93]
	NAFLD vs. C	*BCL2L11* *NAGS* *RMND* *HDHD3*	NA	NA	0.95 0.93 0.98 0.96	[82]
	NAFLD vs. C	*TREM2* *TIMD4*	NA	NA	0.95 0.96	[94]
	NAFLD vs. C	*NDUFA4* *TFAM* *CDKN1B*	NA	NA	0.93 0.90 0.91	[82]
	NAFLD vs. C	*NFE2L2*, *DLD*, and *POLD1*	NA	NA	0.70	[95]
	NAFLD vs. C	*ANXA2*	NA	NA	0.95	[96]
	NASH vs. C	*PHLDA1* *ZFP36L2*	NA	NA	0.78 0.71	[97]
	NAFLD vs. severe NAFLD	*IL32*-ALT-AST	NA	NA	0.92	[30]
	NASH vs. C	*Anax1* and *Gpnmb*	87.5%	59.6%	0.81	[98]

C: control/healthy. F: fibrosis. NA: not applicable.

**Table 2:** MASH/MASLD diagnostic model constructed by proteomic research.

Function	Comparisons	Prediction Models	Sensitivity	Specificity	AUROC	Refs.
lipid metabolism and cell signaling	NASH vs. C	PLIN2-Diabetes-Triglycerides-ALT-waist circumference RAB14	88% 96.9%	100% 34.5%	0.976 0.824	[122]
immunomodulation	simple steatosis vs. MASH	sTREM2	NA	NA	0.8	[111]
	NASH vs. C	sTREM2	54%	89%	0.83	[123]
	NAFL vs. NASH	sTREM2	NA	NA	0.80	[124]
extracellular matrix and cell–cell adhesion	benign steatosis vs. MASH	TSP2	NA	NA	0.84	[125]
metabolic enzyme	NASH vs. C	PTGR1	87%	63%	0.85	[126]
extracellular matrix formation and cell signaling	(1) NAFL/NASH F0–1 vs. NASH F2–3 (2) NAFL/NASH F0–1 vs. at-risk NASH	ADAMTSL2	(1) 58% (2) 68%	(1) 91% (2) 86%	(1) 0.83 (2) 0.86	[127]
cell signaling, extracellular matrix formation, and cell signaling	(1) MASLD vs. C (2) fibrosis stages ≥ 1 vs. C (3) fibrosis stages ≥ 2 vs. C	(1) EPHA10, CILP2, TMEM25, and MPST (2) OGFOD3, ENPP7, and P2RX4 (3) TMEM256 and ICAM1	NA	NA	(1) 0.986 (2) 0.858 (3) 0.837	[128]
cell growth, differentiation, and fibrosis	MASH vs. C	Fibrinogen α, IGFBP-3, COX4-1, HBP1, IGF-1R, VEGFR-2, EGFR, PDGFR-β, and Fibrinogen β	NA	NA	0.79	[129]

C: control/healthy. F: fibrosis. NA: not applicable.

**Table 3:** MASH/MASLD diagnostic model constructed by metabolomic/lipidomic research.

**Metabolite**	**Comparisons**	**Prediction Models**	**Sensitivity**	**Specificity**	**AUROC**	**Refs.**
lipid and fatty acid	High-risk MASH vs. no-risk MASH	MASEF (2 triglycerides, 5 glycerophosphocholines, 1 cholesteryl ester, 1 ceramide, and 3 sphingomyelins)	78%	65%	0.79	[152]
	simple steatosis vs. MASH	OWLiver Panel (OWLiver DM2 and MASEF)	86%	35%	0.788	[153]
	MASLD vs. MASH	DAG 32:1, DAG 34:0, DAG 34:1, TAG 40:1, TAG 44:1, TAG.48:1, TAG 50:2, and SM d36:0	NA	NA	0.808	[155]
	MASLD vs. C	4 HDoHE, 14 HDoHE, 5-HETE, 12-HETE, 15-HETE, 12-HEPE, 5,6-EET, 11,12-EET, 14,15-EET, 15-HETrE, 9,10-diHOME, 9-HODE, DHA, EPA, and adrenic acid	NA	NA	0.999	[154]
	NAFL vs. NASH	FFA (18:0), LPC (22:6/0:0), FFA (18:1), and PI (16:0/18:1)	NA	NA	0.923	[156]
	(1) NAFL vs. C (2) NASH vs. C	HOMA-IR, BMI, platelets count, LDL-c, ferritin, AST, FA 12:0, FA 18:3 ω3, FA 20:4 ω6/FA 20:5 ω3, CAR 4:0, LPC 20:4, LPC O-16:1, LPE 18:0, DG 18:1_18:2, and CE 20:4	(1) 100% (2) 100%	(1) 80% (2) 80%	(1) 0.80 (2) 1.00	[157]
amino acid and lipid	MASLD vs. MASH	glutamate, isoleucine, glycine, lysophosphatidylcholine 16:0, phosphoethanolamine 40:6, AST, and fasting insulin, along with PNPLA3 genotype	86%	35%	0.866	[158]
bile acid	MASH vs. C	Stool GDCA, Stool DCA, Stool GCDCA, Stool TLCA, Stool CDCA, Stool CA, Stool TDCA, Stool LCA, Stool GCA, Stool TCA, Stool TCDCA, Stool GLCA, age, and BMI	NA	NA	0.986	[159]
amino acid	(1) NASH vs. C (2) NAFL vs. NASH	MetaNASH (glutamic acid, isocitric acid, and aspartic acid)	NA	NA	(1) 0.821 (2) 0.719	[160]
organic acid	Mild steatosis vs. severe steatosis	Phenyllactic acid, hydrocinnamic acid, methanobrevibacter, fasting insulin, L-valine, age, 8,11,14-eicosatrienoic acid, suberic acid, BMI, 2-phenylpropionate, HDL, N-acetylserotonin, oxoglutaric acid, N-acetyltryptophan, PWY-3801, PWY-6167, PWY-7345, and Slackia	NA	NA	0.78	[161]

C: control/healthy. NA: not applicable.

## Data Availability

No new data were created or analyzed in this study.
